# The functional basis for variable antipredatory behavioral strategies in the chameleon *Chamaeleo calyptratus*

**DOI:** 10.1242/jeb.242955

**Published:** 2022-05-18

**Authors:** Rachel M. Drown, Andrea L. Liebl, Christopher V. Anderson

**Affiliations:** Department of Biology, University of South Dakota, Vermillion, SD 57069, USA

**Keywords:** Sprint speed, Bite force, Crypsis, Performance, Predation, Chamaeleonidae

## Abstract

To counterbalance demands of different selective pressures, many species possess morphological, physiological and behavioral specializations that increase survival in their environments. Predation is one such pressure that can elicit multiple adaptive responses, and the effectiveness of antipredator behaviors likely vary both by environment and individual across time. Chameleons use multiple antipredator strategies, many of which vary with body size and habitat type. Although their unique morphological and physiological traits produce relatively slow locomotion, which is poorly suited for fleeing, chameleons can also use crypsis or aggression to avoid predation. To examine the functional basis for variable antipredator behavioral responses, we subjected chameleons to a series of mock predation trials and determined how often individuals adopted each antipredator strategy, and then quantified the performance capacities underlying each strategy. In particular, we measured bite force as a determinant for aggression, sprint velocity for fleeing, and degree of color change for crypsis. We found that aggression was predicted by traits associated with higher absolute and relative bite force, as well as habitat type; fleeing was predicted by higher normalized sprint velocity and habitat type; and crypsis was predicted by habitat type, color change capacity in bird color space and the interaction between the two. These results illustrate the importance of considering both functional capacity and environmental context in antipredator behavior decision-making.

## Abstract (precis)

**Summary:** Behavioral antipredator response in chameleons is predicted by both the functional capacity to perform these behaviors and the immediate environmental context for that individual.

## INTRODUCTION

Selective pressures such as predation, resource availability and disease influence all animals. To counterbalance the often-conflicting demands coping with such pressures imposes, many species possess morphological, physiological and behavioral specializations that increase survival in their environment. Predation is well suited for studying the relationship between behavior and environment, as many environmental characteristics (e.g. temperature and the availability of hiding spots) can determine the effectiveness of antipredator behaviors. For example, lower temperatures decrease locomotor performance in reptiles, resulting in lower sprint speeds and reduced capacities to flee in response to a potential threat (Kaufmann and Bennett, 1989; Herrel et al., 2007). Similarly, landscape and vegetation affect the availability of suitable hiding spots, which individuals may occupy when avoiding predator detection. The suite of antipredator behaviors with which an animal is equipped is dependent on multiple factors, which provides a framework to examine the relationships between behavior, environment and morphology/physiology.

As with any trait, there are both costs and benefits associated with every antipredatory behavior. Additionally, the costs and benefits to each strategy may vary based on environment and predator type, resulting in differential use of each behavior depending on the habitat or predator–prey interactions (Stuart-Fox et al., 2008a,b). Fleeing, for instance, is an energetically expensive strategy that may involve relinquishing valuable resources and may also alert predators to an individual's location (Ydenberg and Dill, 1986). In contrast, remaining stationary and cryptic is less energetically expensive than fleeing and may reduce predator attacks by preventing detection (Langridge et al., 2007). However, crypsis is ineffective if the predator manages to detect its cryptic prey. An individual that chooses to aggressively defend itself risks injury by the predator but this strategy may be useful if others are ineffective.

Chameleons are a particularly good model to study the relationship between the environment and antipredator behavior. Their unique morphological and physiological traits, which have adapted in response to their largely arboreal lifestyle, produce relatively slow locomotion, which may be poorly suited for fleeing (Anderson and Higham, 2014; Higham and Anderson, 2014; Herrel, 2014). Compared with other lizards of similar size, chameleons possess lower overall muscle mass, reduced contractile capacities of their locomotor muscles, and a distinct upright limb posture (Abu-Ghalyun et al., 1988). This has resulted in alternative antipredatory strategies that do not require speed (Greene, 1988). Upon detection of a predator, chameleons may flee, but they also might remain motionless while undergoing cryptic color changes or perform aggressive defense behaviors such as lunging and/or biting. Crypsis is a particularly useful strategy in chameleons, as they have exceptional vision and can often detect the predator before the predator detects them (Stuart-Fox, 2014), and can use crypsis to conceal themselves accordingly. With a diversity of antipredatory strategies available to them, the strategy adopted in response to a predator should vary depending on which gives the chameleon its best chance of survival.

The frequency of antipredatory behaviors in chameleons can change across body size, which might reflect variation in life history (Cuadrado et al., 2001). Specifically, juvenile and adult Mediterranean chameleons (*Chamaeleo chamaeleon*) exhibited gaping behavior (an aggressive defense) more frequently than hatchlings when approached by humans in their wild environment, whereas dropping from the branch and free-falling to the ground was displayed more by hatchlings. Additionally, chameleons in a dense habitat allowed closer approach distances by humans (Cuadrado et al., 2001). Whether the capacity to perform specific antipredatory responses relates to the frequency with which those strategies are adopted, however, is unknown.

Rather than size or habitat alone, we hypothesized that variation in which antipredator behavior is employed may also be a result of the chameleons' physiological capability to perform each behavior. To explore this, we quantified the performance capacities underlying the primary antipredator behaviors in chameleons (i.e. bite force for aggression, sprint speed for fleeing, and the degree of color change for crypsis) to examine the functional basis of antipredator behaviors during mock predation trials. We predicted that larger chameleons and chameleons that bit harder for their size would more frequently behave aggressively by lunging and biting during a predation event than smaller individuals and those that bit less hard for their size. We predicted this because the stronger bite forces of these individuals can more effectively ward off a predator through an aggressive response. Further, as velocity is known to be size independent among geometrically similar animals (Hill, 1950), we predicted that smaller chameleons would have higher normalized sprint speeds and therefore rely more on fleeing. Lastly, we expected crypsis to be chosen most often by larger individuals that can adopt a wider range of colors (Teyssier et al., 2015). Although the ability to perform these different behaviors may vary with body size, this functional perspective will help shed light on why individuals of different sizes vary in the frequency of particular behavioral strategies.

## MATERIALS AND METHODS

Male veiled chameleons (*Chamaeleo calyptratus* Duméril & Duméril 1851) were obtained from either introduced populations in Florida, USA, or, in the case of neonate individuals, from commercial breeders. Individuals were housed in glass terrarium enclosures containing live and artificial plants. Enclosures were equipped with fluorescent daylight (6500K) and UVB bulbs, along with an incandescent basking bulb for lighting and temperature regulation. Animals were fed crickets every 2–3 days and provided with water several times a day via an automatic misting system. The use of animals and the experimental protocol for this study were approved by the Institutional Animal Care and Use Committee of the University of South Dakota (protocol no. 06-10-17-20C).

### Behavioral trials

**Fig. 1.**
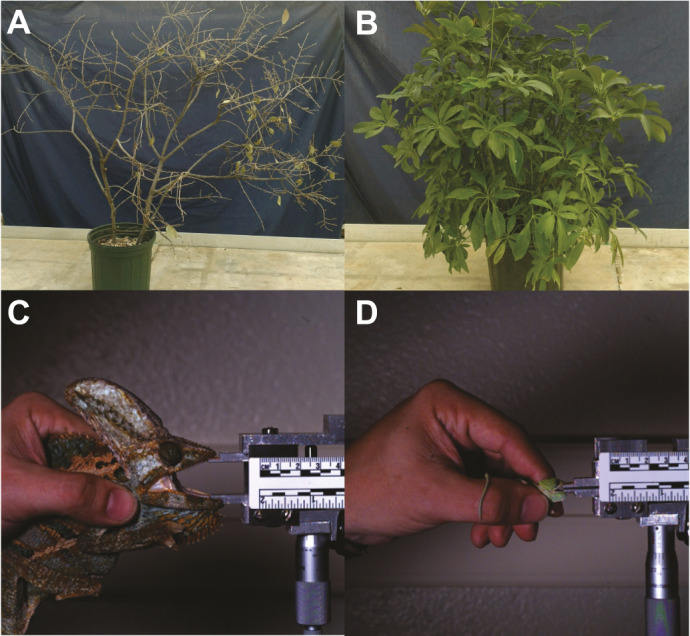
**Experimental habitat and force transducer spacing variation used in experimental trials.** During behavioral trials, chameleons were placed in the center of (A) an open environment consisting of collected branches, or (B) a closed environment containing a dense *Schefflera arboricola* house plant. During bite force performance trials, bite plate spacing was varied based on size classes with bite plates being (C) spaced further apart for larger individuals than (D) smaller individuals.

Each animal was subjected to a total of 16 behavioral trials, at a frequency of one trial every other day for 32 days. Animals were individually placed in the center of alternating experimental arenas simulating (i) an open environment containing sparse vegetation and few hiding spots or (ii) a closed environment with dense vegetation and plentiful hiding spots (Fig. 1A,B). During behavioral trials, animals were presented with either an artificial bird or snake predator model, representing two of the most common chameleon predators (Measey et al., 2014). The combination of environment and predator type for each trial was randomized such that individuals were subjected to four behavioral trials for each of four possible combinations to test for treatment effects on behavior. After a 60 s acclimation period to the environment (longer durations risk the animal attempting to escape from the trial arena), the predator model was marionetted over a curtain that kept the researcher out of the chameleon's view. The snake predator model was made to approach the chameleon from a distance below the chameleon's position within the vegetation, whereas the bird predator model was made to approach from above the chameleon and vegetation to mirror how both predators might approach a vegetation patch. The chameleons were allowed to interact with the predator for 30 s (further durations did not elicit an additional response), with each trial being video recorded for subsequent scoring of behavioral responses. Behavioral responses were categorized as either: (i) aggression (body inflation, mouth gaping, hissing and/or lunging); (ii) fleeing (quick escape from the predator); (iii) crypsis (changing color to more closely match background); (iv) ring-flipping (rotating to the side of the branch opposite from the predator's view; Resetarits and Raxworthy, 2016); (v) free-falling (dropping from the branch); or (vi) leaf-mimicking (slow, back-and-forth movement imitating foliage).

### Performance trials

Within 2 weeks of the completion of behavioral trials, each individual was sequentially subjected to a series of performance trials to quantify bite force, sprint speed and color change capacity, as specified below.

#### Bite force

Maximal bite force was measured as an indicator of an individual's capacity to aggressively defend itself from a predator. Measurements were performed using a piezoelectric isometric force transducer (type 9203, Kistler, Switzerland) incorporated onto a custom-built double-cantilever beam mounting apparatus (Fig. 1C,D; Herrel et al., 1999) and connected to a charge amplifier (type 5010B1, Kistler). Force measurements were sampled at 10 kHz (National Instruments, Austin, TX, USA; Igor Pro 7, Wavemetrics Inc., Lake Oswego, OR, USA). The steel biting surfaces were covered with a small piece of leather to promote more vigorous bites and protect the animals' teeth (Lappin and Jones, 2014). Prior to bite force measurements, the chameleons were placed in an incubator set to 31.7°C, the mean body temperature selected by this species across two studies (Zari, 1993; Andrews, 2008), for 1 h and the distance between the bite plates was adjusted based on the chameleon's size (mean±s.e.m. gape angle of 33.3±1.2 deg across individuals; Fig. 1C,D). Following acclimation to experimental body temperatures, the animals were placed in front of the bite plates, where they were then stimulated by lightly touching around the head and snout to induce biting the bite surfaces of the bite force transducer. Level of stimulation required to elicit bites has been shown to not significantly influence bite force in this species and bite force is very repeatable among trials (Ligon and McGraw, 2018). As a result, four bite trials were collected from each individual, with all trials occurring on the same day. Bite position was normalized across trials according to Lappin and Jones (2014) by filming bites in lateral view and measuring the position of the jaw joint, the tip of the jaw and the point at which they bit down in ImageJ software (National Institutes of Health, Bethesda, MD, USA; http://imagej.nih.gov/ij/). From these measurements, bite force was normalized to a position 50% of the way down the jaw line. The highest normalized bite force measurement from each individual was then used as an approximation of maximal bite performance for that individual (Losos et al., 2002).

#### Sprint speed

The capacity of an individual to flee in response to a predator was measured by calculating sprint performance from position data gathered during sprint trials. As in bite force trials, chameleons were placed in an incubator set to 31.7°C for 1 h prior to the onset of sprint trials. Chameleons were filmed at 500 frames s^−1^ with two high-speed cameras (SC1 Edgertronic high-speed camera, Sanstreak Corp., San Jose, CA, USA) to collect biplanar position data as animals were individually chased down the length of a wooden dowel by an investigator reaching toward the chameleon. Dowels of three different diameters (4.85, 8.65 and 12.65 mm) were used in trials according to the chameleon's size to reduce the influence of substrate material on sprint speed (Losos et al., 1993). Instantaneous body position was measured and calibrated using a digitizing tool (Hedrick, 2008) for MATLAB. A custom script in Igor Pro (Igor Pro 7, Wavemetrics) was used to calculate instantaneous velocity as the first derivative of the calibrated position data, and peak velocity for each sprint trial was extracted from these data.

Peak sprint velocity data were normalized to snout–vent length (SVL). The resultant normalized peak sprint velocity values therefore account for changes in relative position for chameleons of different sizes. These normalized values are relevant from a predation standpoint as smaller chameleons sprinting at the same velocity as large chameleons move more body lengths per second than the larger chameleons. Therefore, smaller prey have reduced positional overlap over any period of time for the predator to target, making it more difficult for a predator to accurately target smaller prey running at the same velocity as larger prey. Five sprint trials were collected per individual on each of three days with a rest day between trial days for 15 total sprint trials per individual. To best approximate maximal sprint performance, only the largest normalized peak velocity from those 15 trials was used for analysis (Losos et al., 2002).

#### Color change capacity

**Fig. 2.**
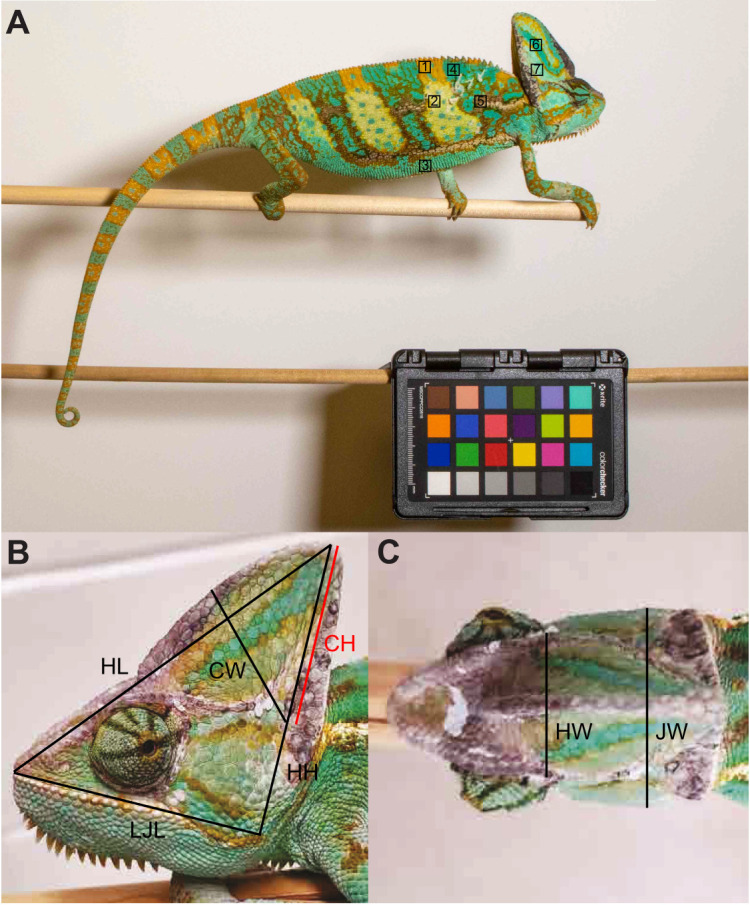
**Color change and cranial dimension analysis landmarks.** Images depicting the (A) locations of seven regions of interest on the head and body of each chameleon that were sampled as part of color change capacity measurements, and (B) lateral and (C) dorsal views of seven cranial dimensions measured to correlate with bite performance. Note the presence of a color standard included in the frame in A, allowing for photos to be linearized and normalized to control for sources of error, including variations in light or exposure. LJL, lower jaw length; HL, head length; HH, head height; CW, casque width; CH, casque height; HW, head width; JW, jaw width.

The degree of color change a chameleon is capable of producing was measured as an indicator of an individual's ability to undergo crypsis. To quantify color change capacity, a series of digital photographs were collected from individuals using a Canon EOS 7D Mark II camera body and a Canon EOS 70–200 mm f/2.8 L IS USM zoom lens. Color change was induced by (i) presenting the chameleon with a predator model; (ii) using a mirror so the chameleon could see its reflection; or (iii) presenting another chameleon approximately 30 cm away. These methods were introduced sequentially until a change of color was produced. All photographs were taken with a color standard (ColorChecker Passport, X-Rite Photo) containing 24 standardized colors of known red, green and blue (RGB) values in the frame (Fig. 2A). These photos were linearized and normalized to control for sources of error, including variation in light or exposure using a toolbox for ImageJ (micaToolbox v2.2; Troscianko and Stevens, 2015). We created cone-catch models within micaToolbox as models for the visual acuity of a passerine bird and a diurnal colubrid snake, two common chameleon predators (Measey et al., 2014), to estimate the detectability of color change to the visual system of both predators ([Bibr JEB242955C28][Bibr JEB242955C29]). To estimate the detectability of color change in passerines, we used an existing spectral sensitivity file for blue tits (*Cyanistes caeruleus*) from micaToolbox. For the colubrid snake, we created spectral sensitivity files for the garter snake (*Thamnophis sirtalis*) based on Sillman et al. (1997) and [Bibr JEB242955C28][Bibr JEB242955C29]) using WebPlotDigitizer v4.5 (https://automeris.io/WebPlotDigitizer/) assuming a ratio of 1:1.6:7.3 for the three snake photoreceptor classes (Vorobyev and Osorio, 1998). The linearized photos of the chameleons were individually converted using these models into color spaces that a bird and a snake predator would perceive. The cone-catch images for both predator types were then converted into receptor noise limited (RNL) chromaticity color space to split the cone-catch values into color channels.

Using previous color analysis methods in chameleons (Ligon and McGraw, 2013; Ligon, 2014), seven regions of interest on the head and body were chosen for these analyses (Fig. 2A). For both the bird and snake color space images, color change was measured in units of just noticeable differences (JNDs) as the Euclidian distance of the mean color channel values in RNL chromaticity color space for each region of interest between sets of photographs of a single individual at the start of the color change procedure and after color change was induced. This produced a JND value of color change for all seven regions of interest on each chameleon in both the bird and snake color space.

### Morphological measurements

Morphological measurements of cranial dimensions, SVL and body mass were collected from each individual at the completion of performance trials. Seven cranial dimensions were measured using digital calipers (±0.01 mm) to explore known relationships between maximal bite force and specific cranial measurements (e.g. Measey et al., 2009, Ligon and McGraw, 2018; Fig. 2B,C): lower jaw length (tip of the snout to the jaw joint), head length (tip of the snout to dorsal tip of the casque), head height (jaw joint to dorsal tip of the casque), casque width (proximal point at the posterior of the casque along the lateral crest where squamosal bone turns dorsally to the midpoint of the casque along the parietal crest), casque height (proximal point of casque to dorsal tip of the casque), head width (distance between proximal points of casque on both sides of the head) and jaw width (distance between outer margins of the jaw joints on both sides of the head). With each chameleon held in an outstretched position, SVL was measured from the tip of the snout to the cloacal opening using digital calipers (±0.01 mm) or, for individuals over 150 mm SVL, a tape measure (±0.1 cm). Finally, body mass was measured using a digital microbalance (±0.001 up to 5 g) or spring scale (±0.5 g between 5 and 60 g or ±2 g over 60 g).

### Statistical analysis

To address collinearity between body size and bite force, test the prediction that individuals that bite harder for their size chose aggression more often, and provide a testable variable of that prediction for inclusion in model selection analyses outlined below, residual values from a general linear model comparing SVL and maximum bite force were calculated as a measure of relative maximum bite force. To summarize color change, two principal component analyses (PCAs) were performed using JND color change values for the seven regions of interest on each individual. The first PCA used JND values from the seven regions of interest in the bird color space, whereas the second PCA used JND values from those regions in the snake color space. Principal component (PC) scores accounting for 20% or greater of the variance were then included in subsequent analyses as measures of color change performance.

General linear models were used to examine the relationship between each of the performance metrics (i.e. bite force, sprint speed and color change capacity) with morphological size, specifically, SVL and cranial measurements. Maximum bite force, SVL and all cranial measurements were log-transformed to meet the assumption of linearity for the general linear models.

We used general linear mixed models with individual as a random factor in the nlme package in R (https://CRAN.R-project.org/package=nlme) to determine how predator type, habitat type, individual performance and SVL, as well as the interactions between predator type and habitat type with SVL and individual performance metrics, influenced which antipredatory behavior individuals used.

We used a backwards model selection approach with Akaike's information criterion corrected for small sample size (AICc) to identify the best fit model(s) (Bolker et al., 2009); models with a difference of <2 between their AICc value and that of the best predictor model (ΔAICc) were treated as equivalent. Relative maximum bite force (residuals of the relationship between SVL and maximum bite force) were used in place of maximum bite force in model selection analyses; this controlled for body size as a confounding factor of bite force measurements in the models and represented a measure of an individual's bite force for their size. Models were created for each of the six behaviors separately. All statistical analyses were conducted using R v. 4.0.3 and all figures were created using ggplot2 (Wickham, 2016).

## RESULTS

Data from a total of 27 male *C. calyptratus* were collected. One individual was excluded from analysis owing to health declines during data collection, which may have impacted behavior and performance data. The remaining 26 individuals ranged in size from 3.4 to 22.6 cm SVL and in mass from 1.26 to 166 g, with 10 individuals ≤9 cm SVL and 7 individuals ≥17 cm SVL (https://github.com/alliebl/Drown-et-al). PCAs of the color change data revealed a single PC score in bird color space (explaining 56.7% of the variation) and two PC scores in snake color space (explaining 28.7% and 21.5% of the variation, respectively).

### Effect of body size on performance

**Fig. 3.**
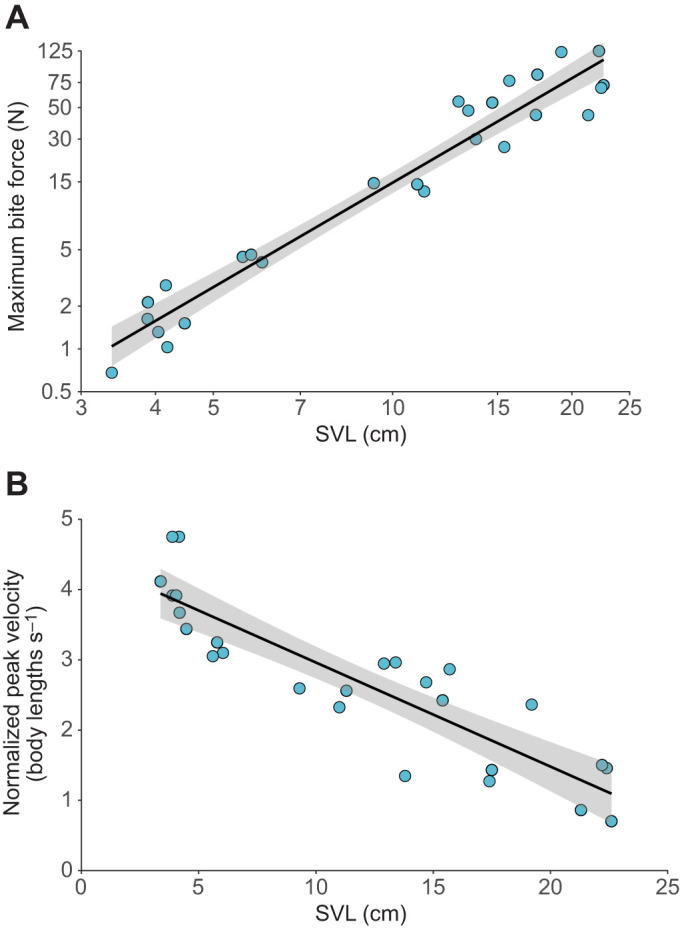
**Body size effects on performance variables.** Relationship between snout–vent length (SVL) and (A) maximum bite force and (B) normalized peak sprint velocity using general linear models. Regression lines are indicated by black lines and shaded areas represent 95% confidence intervals. Note that the relationship in A is plotted on log scales in natural units to linearize exponential relationship, whereas B is plotted on linear scales.

Bite force increased significantly with SVL (Fig. 3A, Table 1), with normalized bite forces ranging from 0.68 to 124.96 N (https://github.com/alliebl/Drown-et-al). Bite force was also positively related to size across all seven cranial measurements (Table 1). The strongest relationship occurred with head length (Table 1).

**Table 1.**
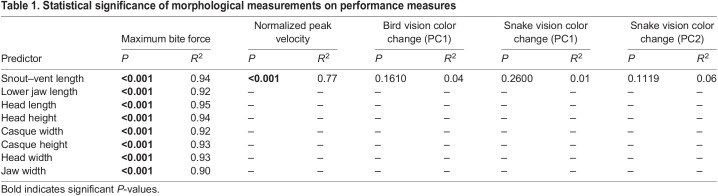
Statistical significance of morphological measurements on performance measures

Normalized peak velocity decreased with increasing SVL (Fig. 3B, Table 1), indicating that smaller individuals ran proportionately faster. No significant relationship between SVL and change of color in either bird or snake color space was observed (Table 1).

### Model selection effects on behavioral responses

**Fig. 4.**
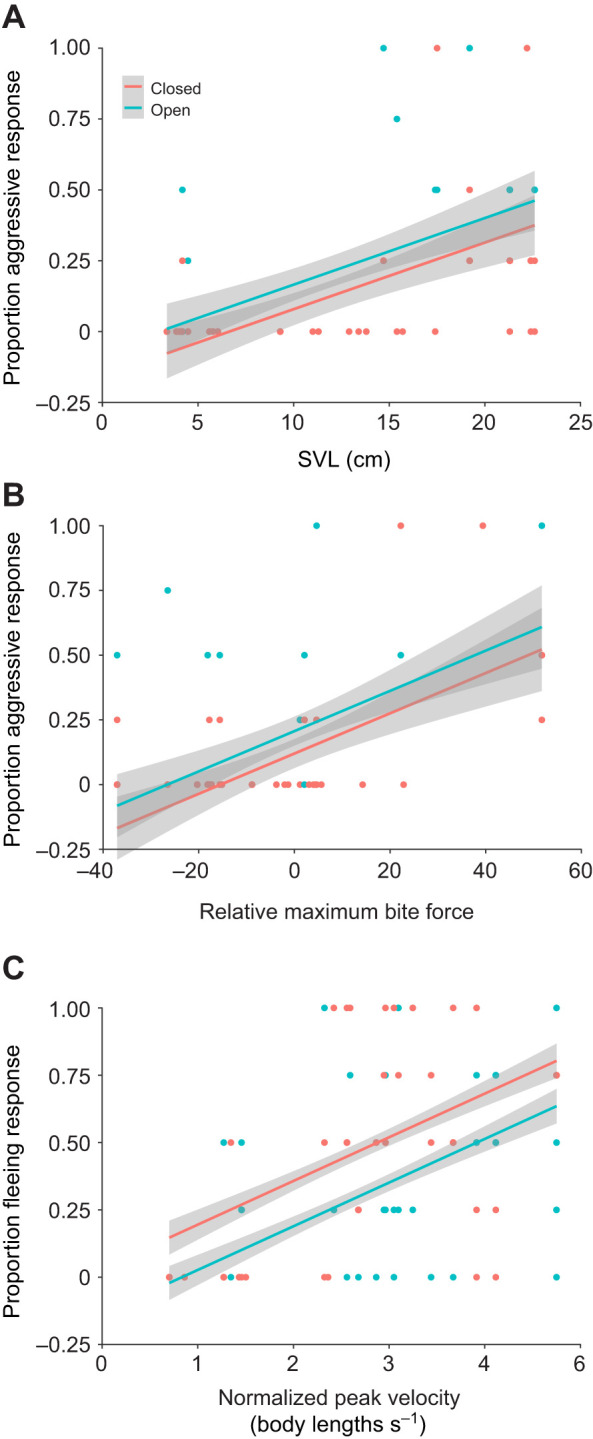
**Predicted fit estimates for model best explaining behavioral choice proportions.** Fit lines represent a general linear mixed model based on predicted values derived from the best-fit model. Individual dots represent proportion of a behavioral choice selected for an individual in one of four habitat and predator combinations (i.e. each individual is represented by four dots: one for trials in an open habitat with a bird predator model, one for trials in an open habitat with a snake predator model, one for trials in a closed habitat with a bird predator model, and one for trials in a closed habitat with a snake predator model). Effect of (A) SVL and (B) relative maximum bite force (residuals) on the proportion of aggressive responses based on a model including habitat, SVL and relative maximum bite force, and the (C) effect of normalized peak velocity on the proportion of fleeing responses based on a model including habitat and normalized peak velocity are shown. The fit relationships in A and B are based on the same general linear mixed model with predicted values calculated based on an average value of (A) relative maximum bite force and (B) SVL. Shaded areas represent 95% confidence intervals.

SVL, habitat and the residuals for the relationship between SVL and maximum bite force (henceforth, relative maximum bite force) were the best predictors for an individual using an aggressive response during mock predation trials (Table 2; Table S1). The aggressive response was chosen more often by larger individuals, by those who bit proportionally harder for their body size, and by individuals in open habitats (Fig. 4A,B).

**Table 2.**
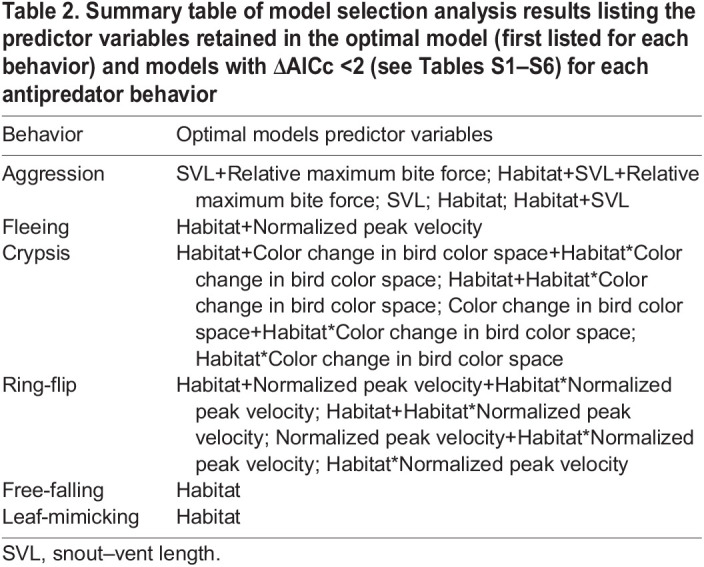
Summary table of model selection analysis results listing the predictor variables retained in the optimal model (first listed for each behavior) and models with ΔAICc <2 (see Tables S1–S6) for each antipredator behavior

Both habitat and normalized peak velocity were retained in the best model predicting a fleeing response (Table 2; Table S2). Individuals with higher normalized peak velocities (running more body lengths per second) chose to flee more frequently than those with lower normalized peak velocities. Further, fleeing was chosen more often in the closed habitat than the open habitat (Fig. 4C).

**Fig. 5.**
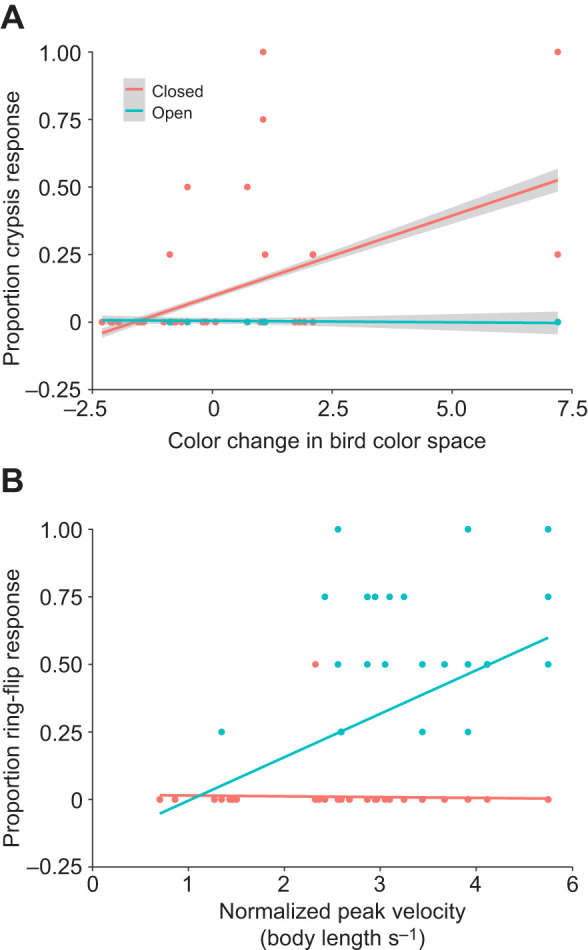
**Predicted fit estimates for models best explaining behavioral choice proportions.** Predicted fit estimate relationships between (A) the capacity to change color as calculated from bird color space and the proportion of crypsis responses based on a model including habitat, color change in bird color space and the interaction between habitat and color change in bird color space, and (B) normalized peak velocity on the proportion of ring-flip responses based on a model including habitat, normalized peak velocity and the interaction between habitat and normalized peak velocity. Fit estimate calculations and indications as in Fig. 4.

Adopting crypsis was best predicted by models containing habitat, color change capacity in the bird color space and the interaction between these two variables (Table 2; Table S3). This behavior was chosen significantly more often among individuals with greater color change capacity in the bird color space when those individuals were in closed environments containing leaves and plentiful hiding spots, but not when those individuals were in open environments containing bare branches (Fig. 5A).

**Fig. 6.**
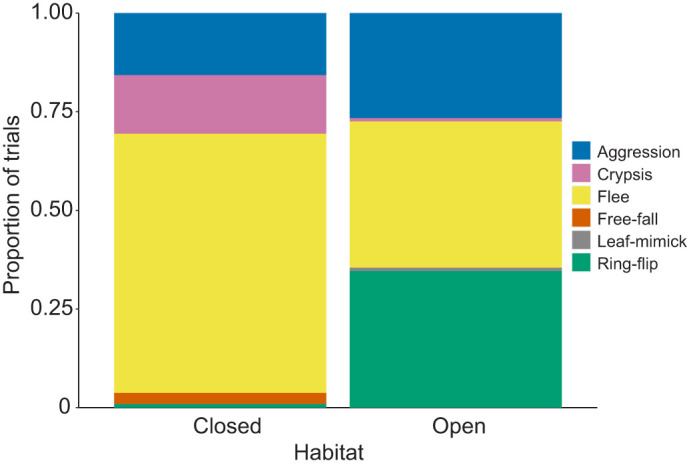
**Relationship between habitat type and proportions of antipredator behavior.** Models including habitat alone significantly predicted free-falling and leaf-mimicking responses.

The ring-flip behavior was best predicted by the model including habitat, normalized peak velocity and the interaction between these two variables. (Table 2; Table S4). Chameleons running at higher normalized peak velocities chose the ring-flip behavior more frequently in open habitats, but not in closed habitats (Fig. 5B). Habitat alone was the best predictor for both free-falling and leaf-mimicking (Table 2; Tables S5,S6), although neither behavior occurred frequently (Fig. 6). Predator type was not retained in any optimal models from the model selection process.

## DISCUSSION

We found that variation in antipredator behavior responses in chameleons can be predicted by differences in an individual's functional capacity to perform them and the environment in which it finds itself during a predation event. In particular, the frequency with which chameleons adopted an aggressive response to a mock predator was best predicted by body size, relative maximum bite force and habitat structure. The frequency with which fleeing was used in response to mock predators was best explained by a combination of the normalized peak sprint velocity and habitat structure. Somewhat similarly, the frequency of active body repositioning (i.e. ‘ring-flip’) was best explained by a combination of the normalized peak sprint velocity, habitat structure and the interaction between these two variables. Finally, the frequency of adopting cryptic color change in response to predation trials, in contrast, was best predicted by a combination of habitat, the capacity to change color as quantified in bird color space and the interaction between these two variables.

The proportion of trials that a chameleon adopted an aggressive response to mock predators was best predicted by models that included both body size (SVL) and relative maximum bite force and habitat (Table 2). Previous work suggested body size alone as a driving factor for choosing aggression, as aggression was chosen most often by larger individuals (Cuadrado et al., 2001). However, our results suggest this behavioral variation may be driven largely by differences in bite force, rather than size itself. Consistent with typical scaling relationships between bite force and size (e.g. Herrel and O'Reilly, 2006), we found a positive relationship between body size and bite force (Fig. 3A) and between body size and the frequency of adopting an aggressive response (Fig. 4A). Further, individuals that bit harder for their body size chose an aggressive response more than individuals of the same size that had a weaker bite force (Fig. 4B). Together, these relationships indicate that a chameleon's physiological capacity to bite, both absolutely and relatively, play a major role in driving patterns of variation in how frequently individuals adopt an aggressive response during predation events. Strong bite forces are likely an extremely effective deterrent in any predation event, resulting in traits related to it driving aggressive responses. Aggression was also chosen more frequently in open habitats (Figs 4A,B and 6), possibly because in open habitats fleeing and crypsis may be less effective strategies.

Similar to aggression being driven by traits related to underlying performance and habitat structure, whether a chameleon chose to flee in response to a mock predator was predicted by both sprint performance and habitat surroundings. In particular, chameleons that were capable of sprinting with higher normalized peak velocities, as well as chameleons located in a closed habitat setting, were more likely to choose to flee during predation trials (Figs 4C and 6). Normalized peak velocity is an indicator of the peak rate of relative positional changes and is therefore particularly relevant from a predation standpoint. Higher normalized peak velocity values correspond with reduced positional overlap over a given time period; this means that individuals with higher normalized peak velocities move out of a specific space faster, reducing the ability of a predator to target a specific location for prey capture. This could be driven largely by body size differences, as a small chameleon moving at the same velocity as a large chameleon moves more body lengths over the same period of time. Body size, however, was not retained in the optimal model predicting fleeing, suggesting that body size differences alone are not driving this pattern with normalized peak velocity. In addition to higher normalized sprint performance, habitat type influenced chameleon fleeing in that chameleons chose to flee more in closed habitats than in open habitats. Together, these patterns suggest that if a chameleon can flee quickly, it may be able to find a safe retreat in denser vegetation, where it might be more difficult for the predator to detect the chameleon or navigate through.

Like fleeing, the ring-flip behavior (i.e. rotating to the side of the branch opposite to the predator) was best predicted by both normalized sprint performance and habitat. An interaction between these two predictors, however, was also retained in all optimal models. Specifically, a ring-flip response was chosen more often by individuals with higher normalized peak velocities when they were in the open environment, but was almost never chosen in a closed environment, regardless of an individual's sprinting performance (Fig. 5B; https://github.com/alliebl/Drown-et-al). The body repositioning associated with this behavior provides a clear relationship with habitat type; it allows the chameleon to drastically reduce its exposed profile, with only their eyes detectable from the sides of the branch, conferring the ability to remain relatively concealed. Consequently, this may be a useful strategy in an environment where crypsis is ineffective, as it still allows them to reduce its detectability. However, the relationship with normalized peak velocity is less clear. Although both ring-flipping and fleeing behaviors were chosen more often by individuals with higher normalized peak velocities, they contrast in which habitat they were used more in. Although fleeing may be effective in a closed environment for relatively fast-running chameleons, in general, chameleons are quite slow-moving. Thus, fleeing in an open environment is likely less effective, and ring-flipping may be a more effective strategy.

Although the degree of color change in both bird and snake color space was quantified, color change in the snake color space was not retained in any model. Rather, models including habitat, color change in bird color space and an interaction between these two predictors best predicted the use of crypsis during predation trials (Table 2). In particular, crypsis was adopted more often by individuals with a greater capacity to change color within the bird color space when they were in a closed habitat, but was almost never chosen in an open habitat (Fig. 5A; https://github.com/alliebl/Drown-et-al). Whereas crypsis is a viable defense option in environments where there is something to camouflage amongst, it would be less effective in sparse habitats. Further, although chameleons are noted to possess differing physiological capabilities for color change throughout ontogeny (Teyssier et al., 2015), with adults having a larger range of colors they can adopt during crypsis, we found no significant relationship between body size and color change capacity in either bird or snake color space. The limited relationships that we observed here may have been caused by insufficiently quantifying and eliciting color change, or by eliciting color change under contexts that differed from their predation response. Chameleon irradiance spectra have UV components, which birds have the ultraviolet photoreceptor spectral sensitivities to detect (Stuart-Fox et al., 2007; Stuart-Fox et al., 2008a,b). However, the camera used to quantify color change was not sensitive to UV light, likely limiting the full spectral visualization from a bird's perspective. Additionally, the ability to detect behavioral response variation in response to changes in an individual's color change capacity in snake color space may have been hindered by the fact that no single PC score encapsulated as much of the variation as was seen in bird color space scores. Further, chameleons change color differently depending on the stimulus (Stuart-Fox et al., 2008a,b), and they also change color for temperature regulation (Walton and Bennett, 1993) and owing to intraspecific interactions (Ligon and McGraw, 2013; Ligon, 2014; Dollion et al., 2020). To induce color change in our experiments, however, a sequence of different and multiple methods was often necessary (i.e. predator, mirror and/or another chameleon). This may have had confounding effects on establishing a stronger relationship between color change performance and behavioral response. The use of a single stimuli and the initiation of color change for a single underlying function across trials, however, is difficult in practice as the chameleons differed considerably in their willingness to change color under different methods. Alternatively, recording color change capacity in response to both snake and bird predator models and including both performance parameters in our models may have avoided some of these confounding effects. Interestingly, no relationship between predator type and the use of crypsis was found, despite previous work showing differences in color responses when chameleons interacted with birds or snakes (Stuart-Fox et al., 2008a,b). That study highlights just how dynamic of a behavioral response crypsis is, with a chameleon able to differentially alter its coloration in response to different stimuli to best remain hidden, a difference we may have better detected using the full avian visual spectrum and/or more specific stimuli during color change performance trials.

Model selection indicated that habitat was the best predictor for the free-falling and leaf-mimicking behaviors. Free-falling may be size dependent, as it has been more frequently observed in hatchlings (Cuadrado et al., 2001), but each of these behavioral responses were chosen fewer than five times here, so an increased sample size may be necessary to determine significant correlative predictors.

No relationships between predator type and antipredator behavior were found. For most of the antipredator behaviors studied here (aggression, fleeing and ring-flip), this is unsurprising as their likelihood of being effective is unlikely to change between predators. For instance, when using aggression to deter a predator, it seems unlikely that a chameleon would bite differently based on the type of predator. Alternatively, it is possible that the manner in which the two different predators were presented during behavioral trials failed to elicit different predator-specific responses and/or only elicited a general antipredator response to sudden movement from an unknown approaching object. The use of particular behaviors in response to predation is likely not an independent decision. For instance, fleeing is likely quite energetically expensive, and individuals may be less apt to run when they have strong bite forces that can effectively ward off a predator. Similarly, smaller chameleons with weaker bite forces may be forced to rely on fleeing despite the energy expenditure it requires to reduce the risk of a close encounter.

### Conclusions

The links between maximal individual performance capacity and the frequency of selecting fleeing, crypsis and aggression in response to predation suggest functional underpinnings to many antipredator behaviors. Maximum performance for bite force varied with body size, and the proportion of the associated antipredator behavior used increased with increased performance. Large chameleons and those with proportionally stronger bite forces for their body size chose aggression more often, and chameleons that ran the fastest relative to their size chose to flee and ring-flip more often than individuals with lower performance. This suggests chameleons are selecting behaviors more frequently that they can better perform, maximizing their chances of avoiding predation. In addition, however, behaviors were also or alternatively habitat-dependent, indicating that chameleons are evaluating their environment and choosing behaviors according to what is most effective in each situation. These results on the relationship between behavior, environment and physiology provide considerable insight into our understanding of the importance of the functional capacity to perform behaviors and environmental context in antipredator behavior decision-making.

## Supplementary Material

Supplementary information
