# Supplementary material for: The functional basis for variable antipredatory behavioral strategies in the chameleon Chamaeleo calyptratus
Source: J Exp Biol. 2022 May 18;225(10):jeb242955. doi: 10.1242/jeb.242955 (PMC9167576; doi:10.1242/jeb.242955)
Supplement: Supplementary information [file jexbio-225-242955-s1.pdf]

Table S1. Model selection using Akaike's information criterion corrected for small sample size (AICc) for aggression. Individual was treated as a random factor. Lower AICc values are indicative of better model predictive power. Model K indicates the number of predictors used in the model, ΔAICc represents difference between a model's AICc value and that of the best predictor model, and wi is the AICc weight. Models with a ΔAICc <2 are denoted in bold.

Aggression Models	K	AICc	ΔAICc	wi
**SVL + Relative Maximum Bite Force**	2	4.14	**0.00**	0.28
**Habitat + SVL + Relative Maximum Bite Force**	3	4.94	**0.79**	0.19
**SVL**	1	5.12	**0.98**	0.17
**Habitat**	1	5.29	**1.14**	0.16
**Habitat + SVL**	2	5.87	**1.73**	0.12
Relative Maximum Bite Force	1	8.08	3.94	0.04
Habitat + Relative Maximum Bite Force	2	8.83	4.69	0.03
Habitat + SVL + Normalized Peak Velocity + Relative Maximum Bite Force	4	9.39	5.25	0.02
Habitat + SVL + Normalized Peak Velocity + Bird Color Space PC1 + Relative Maximum Bite Force	5	17.77	13.63	0.00
Habitat + SVL + Normalized Peak Velocity + Bird Color Space PC1 + Snake Color Space PC1 + Relative Maximum Bite Force	6	24.36	20.22	0.00
Habitat + SVL + Normalized Peak Velocity + Bird Color Space PC1 + Snake Color Space PC1 + Snake Color Space PC2 + Relative Maximum Bite Force	7	27.20	23.06	0.00
Habitat + SVL + Predator + Normalized Peak Velocity + Bird Color Space PC1 + Snake Color Space PC1 + Snake Color Space PC2 + Relative Maximum Bite Force	8	33.91	29.77	0.00
Habitat + SVL + Predator + Normalized Peak Velocity + Bird Color Space PC1 + Snake Color Space PC1 + Snake Color Space PC2 + Relative Maximum Bite Force + Habitat*SVL	9	43.32	39.18	0.00
Habitat + SVL + Predator + Normalized Peak Velocity + Bird Color Space PC1 + Snake Color Space PC1 + Snake Color Space PC2 + Relative Maximum Bite Force + Habitat*SVL + Habitat*Normalized	10	46.54	42.40	0.00

Journal of Experimental Biology • Supplementary information

Peak Velocity				
Habitat + SVL + Predator + Normalized Peak Velocity + Bird Color Space PC1 + Snake Color Space PC1 + Snake Color Space PC2 + Relative Maximum Bite Force + Habitat*SVL + Habitat*Normalized Peak Velocity + Habitat*Bird Color Space PC1	11	55.35	51.21	0.00
Habitat + SVL + Predator + Normalized Peak Velocity + Bird Color Space PC1 + Snake Color Space PC1 + Snake Color Space PC2 + Relative Maximum Bite Force + Habitat*SVL + Habitat*Normalized Peak Velocity + Habitat*Bird Color Space PC1 + Habitat*Snake Color Space PC1	12	62.47	58.32	0.00
Habitat + SVL + Predator + Normalized Peak Velocity + Bird Color Space PC1 + Snake Color Space PC1 + Snake Color Space PC2 + Relative Maximum Bite Force + Habitat*SVL + Habitat*Normalized Peak Velocity + Habitat*Bird Color Space PC1 + Habitat*Snake Color Space PC1 + Habitat*Snake Color Space PC2	13	66.42	62.28	0.00
Habitat + SVL + Predator + Normalized Peak Velocity + Bird Color Space PC1 + Snake Color Space PC1 + Snake Color Space PC2 + Relative Maximum Bite Force + Habitat*SVL + Habitat*Normalized Peak Velocity + Habitat*Bird Color Space PC1 + Habitat*Snake Color Space PC1 + Habitat*Snake Color Space PC2 + Habitat*Relative Maximum Bite Force	14	79.41	75.27	0.00
Habitat + SVL + Predator + Normalized Peak Velocity + Bird Color Space PC1 + Snake Color Space PC1 + Snake Color Space PC2 + Relative Maximum Bite Force + Habitat*SVL + Habitat*Normalized Peak Velocity + Habitat*Bird Color Space PC1 + Habitat*Snake Color Space PC1 + Habitat*Snake Color Space PC2 + Habitat*Relative Maximum Bite Force + Predator*SVL	15	90.98	86.84	0.00
Habitat + SVL + Predator + Normalized Peak Velocity + Bird Color Space PC1 + Snake Color Space PC1 + Snake Color Space PC2 + Relative Maximum Bite Force + Habitat*SVL + Habitat*Normalized Peak Velocity + Habitat*Bird Color Space PC1 + Habitat*Snake Color Space PC1 + Habitat*Snake Color Space PC2 + Habitat*Relative Maximum Bite Force + Predator*SVL + Predator*Normalized Peak Velocity	16	97.63	93.49	0.00
Habitat + SVL + Predator + Normalized Peak Velocity + Bird Color Space PC1 + Snake Color Space PC1 + Snake Color Space PC2 +	17	104.27	100.13	0.00

Journal of Experimental Biology • Supplementary information

Relative Maximum Bite Force + Habitat*SVL + Habitat*Normalized Peak Velocity + Habitat*Bird Color Space PC1 + Habitat*Snake Color Space PC1 + Habitat*Snake Color Space PC2 + Habitat*Relative Maximum Bite Force + Predator*SVL + Predator*Normalized Peak Velocity + Predator*Bird Color Space PC1				
Habitat + SVL + Predator + Normalized Peak Velocity + Bird Color Space PC1 + Snake Color Space PC1 + Snake Color Space PC2 + Relative Maximum Bite Force + Habitat*SVL + Habitat*Normalized Peak Velocity + Habitat*Bird Color Space PC1 + Habitat*Snake Color Space PC1 + Habitat*Snake Color Space PC2 + Habitat*Relative Maximum Bite Force + Predator*SVL + Predator*Normalized Peak Velocity + Predator*Bird Color Space PC1 + Predator*Snake Color Space PC1	18	111.75	107.60	0.00
Habitat + SVL + Predator + Normalized Peak Velocity + Bird Color Space PC1 + Snake Color Space PC1 + Snake Color Space PC2 + Relative Maximum Bite Force + Habitat*SVL + Habitat*Normalized Peak Velocity + Habitat*Bird Color Space PC1 + Habitat*Snake Color Space PC1 + Habitat*Snake Color Space PC2 + Habitat*Relative Maximum Bite Force + Predator*SVL + Predator*Normalized Peak Velocity + Predator*Bird Color Space PC1 + Predator*Snake Color Space PC1 + Predator*Snake Color Space PC2	19	119.14	114.99	0.00
Habitat + SVL + Predator + Normalized Peak Velocity + Bird Color Space PC1 + Snake Color Space PC1 + Snake Color Space PC2 + Relative Maximum Bite Force + Habitat*SVL + Habitat*Normalized Peak Velocity + Habitat*Bird Color Space PC1 + Habitat*Snake Color Space PC1 + Habitat*Snake Color Space PC2 + Habitat*Relative Maximum Bite Force + Predator*SVL + Predator*Normalized Peak Velocity + Predator*Bird Color Space PC1 + Predator*Snake Color Space PC1 + Predator*Snake Color Space PC2 + Predator*Relative Maximum Bite Force	20	132.86	128.72	0.00

Journal of Experimental Biology • Supplementary information

Table S2. AICc model selection for fleeing behavior. Individual was treated as a random factor. Abbreviations as in Supplementary Table 3. Models with a ΔAICc <2 are denoted in bold.

Fleeing Models	K	AICc	ΔAICc	wi
**Habitat + Normalized Peak Velocity**	2	79.14	**0.00**	0.72
Normalized Peak Velocity	1	81.37	2.23	0.23
Habitat + SVL + Normalized Peak Velocity	3	85.47	6.33	0.03
Habitat	1	86.40	7.26	0.02
Habitat + SVL + Normalized Peak Velocity + Relative Maximum Bite Force	4	95.53	16.39	0.00
Habitat + SVL + Predator + Normalized Peak Velocity + Relative Maximum Bite Force	5	101.70	22.56	0.00
Habitat + SVL + Predator + Normalized Peak Velocity + Bird Color Space PC1 + Relative Maximum Bite Force	6	109.67	30.53	0.00
Habitat + SVL + Predator + Normalized Peak Velocity + Bird Color Space PC1 + Snake Color Space PC1 + Relative Maximum Bite Force	7	115.26	36.12	0.00
Habitat + SVL + Predator + Normalized Peak Velocity + Bird Color Space PC1 + Snake Color Space PC1 + Snake Color Space PC2 + Relative Maximum Bite Force	8	122.38	43.24	0.00
Habitat + SVL + Predator + Normalized Peak Velocity + Bird Color Space PC1 + Snake Color Space PC1 + Snake Color Space PC2 + Relative Maximum Bite Force + Habitat*SVL	9	129.11	49.97	0.00
Habitat + SVL + Predator + Normalized Peak Velocity + Bird Color Space PC1 + Snake Color Space PC1 + Snake Color Space PC2 + Relative Maximum Bite Force + Habitat*SVL + Habitat*Normalized Peak Velocity	10	134.19	55.05	0.00
Habitat + SVL + Predator + Normalized Peak Velocity + Bird Color Space PC1 + Snake Color Space PC1 + Snake Color Space PC2 + Relative Maximum Bite Force + Habitat*SVL + Habitat*Normalized Peak Velocity + Habitat*Bird Color Space PC1	11	139.36	60.22	0.00
Habitat + SVL + Predator + Normalized Peak Velocity + Bird Color Space PC1 + Snake Color Space PC1 + Snake Color Space PC2 + Relative Maximum Bite Force + Habitat*SVL + Habitat*Normalized Peak	12	145.37	66.23	0.00

Journal of Experimental Biology • Supplementary information

Velocity + Habitat*Bird Color Space PC1 + Habitat*Snake Color Space PC1				
Habitat + SVL + Predator + Normalized Peak Velocity + Bird Color Space PC1 + Snake Color Space PC1 + Snake Color Space PC2 + Relative Maximum Bite Force + Habitat*SVL + Habitat*Normalized Peak Velocity + Habitat*Bird Color Space PC1 + Habitat*Snake Color Space PC1 + Habitat*Snake Color Space PC2	13	150.90	71.76	0.00
Habitat + SVL + Predator + Normalized Peak Velocity + Bird Color Space PC1 + Snake Color Space PC1 + Snake Color Space PC2 + Relative Maximum Bite Force + Habitat*SVL + Habitat*Normalized Peak Velocity + Habitat*Bird Color Space PC1 + Habitat*Snake Color Space PC1 + Habitat*Snake Color Space PC2 + Habitat*Relative Maximum Bite Force	14	163.16	84.02	0.00
Habitat + SVL + Predator + Normalized Peak Velocity + Bird Color Space PC1 + Snake Color Space PC1 + Snake Color Space PC2 + Relative Maximum Bite Force + Habitat*SVL + Habitat*Normalized Peak Velocity + Habitat*Bird Color Space PC1 + Habitat*Snake Color Space PC1 + Habitat*Snake Color Space PC2 + Habitat*Relative Maximum Bite Force + Predator*SVL	15	173.70	94.56	0.00
Habitat + SVL + Predator + Normalized Peak Velocity + Bird Color Space PC1 + Snake Color Space PC1 + Snake Color Space PC2 + Relative Maximum Bite Force + Habitat*SVL + Habitat*Normalized Peak Velocity + Habitat*Bird Color Space PC1 + Habitat*Snake Color Space PC1 + Habitat*Snake Color Space PC2 + Habitat*Relative Maximum Bite Force + Predator*SVL + Predator*Normalized Peak Velocity	16	179.26	100.12	0.00
Habitat + SVL + Predator + Normalized Peak Velocity + Bird Color Space PC1 + Snake Color Space PC1 + Snake Color Space PC2 + Relative Maximum Bite Force + Habitat*SVL + Habitat*Normalized Peak Velocity + Habitat*Bird Color Space PC1 + Habitat*Snake Color Space PC1 + Habitat*Snake Color Space PC2 + Habitat*Relative Maximum Bite Force + Predator*SVL + Predator*Normalized Peak Velocity + Predator*Bird Color Space PC1	17	186.53	107.39	0.00
Habitat + SVL + Predator + Normalized Peak Velocity + Bird Color Space PC1 + Snake Color Space PC1 + Snake Color Space PC2 + Relative Maximum Bite Force + Habitat*SVL + Habitat*Normalized Peak Velocity + Habitat*Bird Color Space PC1 + Habitat*Snake Color Space	18	192.53	113.39	0.00

PC1 + Habitat*Snake Color Space PC2 + Habitat*Relative Maximum Bite Force + Predator*SVL + Predator*Normalized Peak Velocity + Predator*Bird Color Space PC1 + Predator*Snake Color Space PC1				
Habitat + SVL + Predator + Normalized Peak Velocity + Bird Color Space PC1 + Snake Color Space PC1 + Snake Color Space PC2 + Relative Maximum Bite Force + Habitat*SVL + Habitat*Normalized Peak Velocity + Habitat*Bird Color Space PC1 + Habitat*Snake Color Space PC1 + Habitat*Snake Color Space PC2 + Habitat*Relative Maximum Bite Force + Predator*SVL + Predator*Normalized Peak Velocity + Predator*Bird Color Space PC1 + Predator*Snake Color Space PC1 + Predator*Snake Color Space PC2	19	199.80	120.66	0.00
Habitat + SVL + Predator + Normalized Peak Velocity + Bird Color Space PC1 + Snake Color Space PC1 + Snake Color Space PC2 + Relative Maximum Bite Force + Habitat*SVL + Habitat*Normalized Peak Velocity + Habitat*Bird Color Space PC1 + Habitat*Snake Color Space PC1 + Habitat*Snake Color Space PC2 + Habitat*Relative Maximum Bite Force + Predator*SVL + Predator*Normalized Peak Velocity + Predator*Bird Color Space PC1 + Predator*Snake Color Space PC1 + Predator*Snake Color Space PC2 + Predator*Relative Maximum Bite Force	20	212.39	133.25	0.00

Journal of Experimental Biology • Supplementary information

Table S3. AICc model selection for crypsis behavior. Individual was treated as a random factor. Abbreviations as in Supplementary Table 3. Models with a ΔAICc <2 are denoted in bold.

Crypsis Models	K	AICc	ΔAICc	wi
**Habitat + Bird Color Space PC1 + Habitat*Bird Color Space PC1**	3	-67.75	**0.00**	0.25
**Habitat + Habitat*Bird Color Space PC1**	2	-67.75	**0.00**	0.25
**Bird Color Space PC1 + Habitat*Bird Color Space PC1**	2	-67.75	**0.00**	0.25
**Habitat*Bird Color Space PC1**	1	-67.75	**0.00**	0.25
Habitat	1	-60.33	7.42	0.01
Habitat + Bird Color Space PC1	2	-59.25	8.50	0.00
Habitat + SVL + Bird Color Space PC1 + Habitat*Bird Color Space PC1	4	-57.98	9.76	0.00
Bird Color Space PC1	1	-57.76	9.99	0.00
Habitat + SVL + Normalized Peak Velocity + Bird Color Space PC1 + Habitat*Bird Color Space PC1	5	-50.78	16.97	0.00
Habitat + SVL + Predator + Normalized Peak Velocity + Bird Color Space PC1 + Habitat*Bird Color Space PC1	6	-44.55	23.20	0.00
Habitat + SVL + Predator + Normalized Peak Velocity + Bird Color Space PC1 + Snake Color Space PC1 + Habitat*Bird Color Space PC1	7	-36.49	31.26	0.00
Habitat + SVL + Predator + Normalized Peak Velocity + Bird Color Space PC1 + Snake Color Space PC1 + Snake Color Space PC2 + Habitat*Bird Color Space PC1	8	-27.60	40.14	0.00
Habitat + SVL + Predator + Normalized Peak Velocity + Bird Color Space PC1 + Snake Color Space PC1 + Snake Color Space PC2 + Relative Maximum Bite Force + Habitat*Bird Color Space PC1	9	-13.51	54.24	0.00
Habitat + SVL + Predator + Normalized Peak Velocity + Bird Color Space PC1 + Snake Color Space PC1 + Snake Color Space PC2 + Relative Maximum Bite Force + Habitat*SVL + Habitat*Bird Color Space PC1	10	-5.72	62.02	0.00
Habitat + SVL + Predator + Normalized Peak Velocity + Bird Color Space PC1 + Snake Color Space PC1 + Snake Color Space PC2 + Relative Maximum Bite Force + Habitat*SVL + Habitat*Normalized	11	0.88	68.63	0.00

Peak Velocity + Habitat*Bird Color Space PC1				
Habitat + SVL + Predator + Normalized Peak Velocity + Bird Color Space PC1 + Snake Color Space PC1 + Snake Color Space PC2 + Relative Maximum Bite Force + Habitat*SVL + Habitat*Normalized Peak Velocity + Habitat*Bird Color Space PC1 + Habitat*Snake Color Space PC1	12	8.45	76.20	0.00
Habitat + SVL + Predator + Normalized Peak Velocity + Bird Color Space PC1 + Snake Color Space PC1 + Snake Color Space PC2 + Relative Maximum Bite Force + Habitat*SVL + Habitat*Normalized Peak Velocity + Habitat*Bird Color Space PC1 + Habitat*Snake Color Space PC1 + Habitat*Snake Color Space PC2	13	16.91	84.66	0.00
Habitat + SVL + Predator + Normalized Peak Velocity + Bird Color Space PC1 + Snake Color Space PC1 + Snake Color Space PC2 + Relative Maximum Bite Force + Habitat*SVL + Habitat*Normalized Peak Velocity + Habitat*Bird Color Space PC1 + Habitat*Snake Color Space PC1 + Habitat*Snake Color Space PC2 + Habitat*Relative Maximum Bite Force	14	30.41	98.15	0.00
Habitat + SVL + Predator + Normalized Peak Velocity + Bird Color Space PC1 + Snake Color Space PC1 + Snake Color Space PC2 + Relative Maximum Bite Force + Habitat*SVL + Habitat*Normalized Peak Velocity + Habitat*Bird Color Space PC1 + Habitat*Snake Color Space PC1 + Habitat*Snake Color Space PC2 + Habitat*Relative Maximum Bite Force + Predator*SVL	15	39.95	107.70	0.00
Habitat + SVL + Predator + Normalized Peak Velocity + Bird Color Space PC1 + Snake Color Space PC1 + Snake Color Space PC2 + Relative Maximum Bite Force + Habitat*SVL + Habitat*Normalized Peak Velocity + Habitat*Bird Color Space PC1 + Habitat*Snake Color Space PC1 + Habitat*Snake Color Space PC2 + Habitat*Relative Maximum Bite Force + Predator*SVL + Predator*Normalized Peak Velocity	16	46.85	114.60	0.00
Habitat + SVL + Predator + Normalized Peak Velocity + Bird Color Space PC1 + Snake Color Space PC1 + Snake Color Space PC2 + Relative Maximum Bite Force + Habitat*SVL + Habitat*Normalized Peak Velocity + Habitat*Bird Color Space PC1 + Habitat*Snake Color Space PC1 + Habitat*Snake Color Space PC2 + Habitat*Relative Maximum Bite Force + Predator*SVL + Predator*Normalized Peak	17	53.76	121.51	0.00

Journal of Experimental Biology • Supplementary information

Velocity + Predator*Bird Color Space PC1				
Habitat + SVL + Predator + Normalized Peak Velocity + Bird Color Space PC1 + Snake Color Space PC1 + Snake Color Space PC2 + Relative Maximum Bite Force + Habitat*SVL + Habitat*Normalized Peak Velocity + Habitat*Bird Color Space PC1 + Habitat*Snake Color Space PC1 + Habitat*Snake Color Space PC2 + Habitat*Relative Maximum Bite Force + Predator*SVL + Predator*Normalized Peak Velocity + Predator*Bird Color Space PC1 + Predator*Snake Color Space PC1	18	61.73	129.47	0.00
Habitat + SVL + Predator + Normalized Peak Velocity + Bird Color Space PC1 + Snake Color Space PC1 + Snake Color Space PC2 + Relative Maximum Bite Force + Habitat*SVL + Habitat*Normalized Peak Velocity + Habitat*Bird Color Space PC1 + Habitat*Snake Color Space PC1 + Habitat*Snake Color Space PC2 + Habitat*Relative Maximum Bite Force + Predator*SVL + Predator*Normalized Peak Velocity + Predator*Bird Color Space PC1 + Predator*Snake Color Space PC1 + Predator*Snake Color Space PC2	19	70.33	138.08	0.00
Habitat + SVL + Predator + Normalized Peak Velocity + Bird Color Space PC1 + Snake Color Space PC1 + Snake Color Space PC2 + Relative Maximum Bite Force + Habitat*SVL + Habitat*Normalized Peak Velocity + Habitat*Bird Color Space PC1 + Habitat*Snake Color Space PC1 + Habitat*Snake Color Space PC2 + Habitat*Relative Maximum Bite Force + Predator*SVL + Predator*Normalized Peak Velocity + Predator*Bird Color Space PC1 + Predator*Snake Color Space PC1 + Predator*Snake Color Space PC2 + Predator*Relative Maximum Bite Force	20	84.53	152.28	0.00

Journal of Experimental Biology • Supplementary information

Table S4. AICc model selection for the ring-flip behavior. Individual was treated as a random factor. Abbreviations as in Supplementary Table 3. Models with a ΔAICc <2 are denoted in bold.

Ring-flip Models	K	AICc	ΔAICc	wi
**Habitat + Normalized Peak Velocity + Habitat*Normalized Peak Velocity**	3	-4.02	**0.00**	0.25
**Habitat + Habitat*Normalized Peak Velocity**	2	-4.02	**0.00**	0.25
**Normalized Peak Velocity + Habitat*Normalized Peak Velocity**	2	-4.02	**0.00**	0.25
**Habitat*Normalized Peak Velocity**	1	-4.02	**0.00**	0.25
Habitat + SVL + Normalized Peak Velocity + Habitat*Normalized Peak Velocity	4	5.35	9.37	0.00
Habitat + Normalized Peak Velocity	2	7.59	11.61	0.00
Habitat + SVL + Predator + Normalized Peak Velocity + Habitat*Normalized Peak Velocity	5	12.23	16.25	0.00
Habitat	1	13.12	17.14	0.00
Habitat + SVL + Predator + Normalized Peak Velocity + Bird Color Space PC1 + Habitat*Normalized Peak Velocity	6	19.23	23.25	0.00
Habitat + SVL + Predator + Normalized Peak Velocity + Bird Color Space PC1 + Snake Color Space PC1 + Habitat*Normalized Peak Velocity	7	26.52	30.54	0.00
Normalized Peak Velocity	1	31.30	35.32	0.00
Habitat + SVL + Predator + Normalized Peak Velocity + Bird Color Space PC1 + Snake Color Space PC1 + Snake Color Space PC2 + Habitat*Normalized Peak Velocity	8	35.11	39.13	0.00
Habitat + SVL + Predator + Normalized Peak Velocity + Bird Color Space PC1 + Snake Color Space PC1 + Snake Color Space PC2 + Relative Maximum Bite Force + Habitat*Normalized Peak Velocity	9	48.66	52.68	0.00
Habitat + SVL + Predator + Normalized Peak Velocity + Bird Color Space PC1 + Snake Color Space PC1 + Snake Color Space PC2 + Relative Maximum Bite Force + Habitat*SVL + Habitat*Normalized Peak Velocity	10	57.68	61.70	0.00
Habitat + SVL + Predator + Normalized Peak Velocity + Bird Color Space PC1 + Snake Color Space PC1 + Snake Color Space PC2 + Relative Maximum Bite Force + Habitat*SVL + Habitat*Normalized Peak Velocity + Habitat*Bird Color Space PC1	11	64.30	68.32	0.00
Habitat + SVL + Predator + Normalized Peak Velocity + Bird Color Space PC1 + Snake Color Space PC1 + Snake Color Space PC2 + Relative Maximum Bite Force + Habitat*SVL + Habitat*Normalized Peak Velocity + Habitat*Bird Color Space PC1 + Habitat*Snake Color Space PC1	12	70.07	74.08	0.00
Habitat + SVL + Predator + Normalized Peak Velocity + Bird Color Space PC1 + Snake Color Space PC1 +	13	77.63	81.65	0.00

Snake Color Space PC2 + Relative Maximum Bite Force + Habitat*SVL + Habitat*Normalized Peak Velocity + Habitat*Bird Color Space PC1 + Habitat*Snake Color Space PC1 + Habitat*Snake Color Space PC2				
Habitat + SVL + Predator + Normalized Peak Velocity + Bird Color Space PC1 + Snake Color Space PC1 + Snake Color Space PC2 + Relative Maximum Bite Force + Habitat*SVL + Habitat*Normalized Peak Velocity + Habitat*Bird Color Space PC1 + Habitat*Snake Color Space PC1 + Habitat*Snake Color Space PC2 + Habitat*Relative Maximum Bite Force	14	90.47	94.49	0.00
Habitat + SVL + Predator + Normalized Peak Velocity + Bird Color Space PC1 + Snake Color Space PC1 + Snake Color Space PC2 + Relative Maximum Bite Force + Habitat*SVL + Habitat*Normalized Peak Velocity + Habitat*Bird Color Space PC1 + Habitat*Snake Color Space PC1 + Habitat*Snake Color Space PC2 + Habitat*Relative Maximum Bite Force + Predator*SVL	15	100.74	104.76	0.00
Habitat + SVL + Predator + Normalized Peak Velocity + Bird Color Space PC1 + Snake Color Space PC1 + Snake Color Space PC2 + Relative Maximum Bite Force + Habitat*SVL + Habitat*Normalized Peak Velocity + Habitat*Bird Color Space PC1 + Habitat*Snake Color Space PC1 + Habitat*Snake Color Space PC2 + Habitat*Relative Maximum Bite Force + Predator*SVL + Predator*Normalized Peak Velocity	16	106.62	110.64	0.00
Habitat + SVL + Predator + Normalized Peak Velocity + Bird Color Space PC1 + Snake Color Space PC1 + Snake Color Space PC2 + Relative Maximum Bite Force + Habitat*SVL + Habitat*Normalized Peak Velocity + Habitat*Bird Color Space PC1 + Habitat*Snake Color Space PC1 + Habitat*Snake Color Space PC2 + Habitat*Relative Maximum Bite Force + Predator*SVL + Predator*Normalized Peak Velocity + Predator*Bird Color Space PC1	17	114.84	118.86	0.00
Habitat + SVL + Predator + Normalized Peak Velocity + Bird Color Space PC1 + Snake Color Space PC1 + Snake Color Space PC2 + Relative Maximum Bite Force + Habitat*SVL + Habitat*Normalized Peak Velocity + Habitat*Bird Color Space PC1 + Habitat*Snake Color Space PC1 + Habitat*Snake Color Space PC2 + Habitat*Relative Maximum Bite Force + Predator*SVL + Predator*Normalized Peak Velocity + Predator*Bird Color Space PC1 + Predator*Snake Color Space PC1	18	121.61	125.63	0.00
Habitat + SVL + Predator + Normalized Peak Velocity + Bird Color Space PC1 + Snake Color Space PC1 + Snake Color Space PC2 + Relative Maximum Bite Force + Habitat*SVL + Habitat*Normalized Peak Velocity + Habitat*Bird Color Space PC1 + Habitat*Snake Color Space PC1 + Habitat*Snake Color Space PC2 + Habitat*Relative Maximum Bite Force + Predator*SVL + Predator*Normalized Peak Velocity + Predator*Bird Color Space PC1 + Predator*Snake Color Space PC1 + Predator*Snake Color Space PC2	19	125.82	129.83	0.00
Habitat + SVL + Predator + Normalized Peak Velocity + Bird Color Space PC1 + Snake Color Space PC1 + Snake Color Space PC2 + Relative Maximum Bite Force + Habitat*SVL + Habitat*Normalized Peak Velocity + Habitat*Bird Color Space PC1 + Habitat*Snake Color Space PC1 + Habitat*Snake Color Space PC2 + Habitat*Relative Maximum Bite Force + Predator*SVL + Predator*Normalized Peak Velocity + Predator*Bird Color Space PC1 + Predator*Snake Color Space PC1 + Predator*Snake Color Space PC2 + Predator*Relative Maximum Bite Force	20	138.97	142.98	0.00

Table S5. AICc model selection for free-falling behaviors. Individual was treated as a random factor. Abbreviations as in Supplementary Table 3. Models with a ΔAICc <2 are denoted in bold.

Free-falling Models	K	AICc	ΔAICc	wi
**Habitat**	1	-342.78	**0.00**	1.00
Habitat + SVL	2	-329.23	13.55	0.00
Habitat + SVL + Normalized Peak Velocity	3	-319.27	23.51	0.00
Habitat + SVL + Normalized Peak Velocity + Relative Maximum Bite Force	4	-302.43	40.35	0.00
Habitat + SVL + Predator + Normalized Peak Velocity + Relative Maximum Bite Force	5	-292.66	50.12	0.00
Habitat + SVL + Predator + Normalized Peak Velocity + Bird Color Space PC1 + Relative Maximum Bite Force	6	-279.96	62.82	0.00
Habitat + SVL + Predator + Normalized Peak Velocity + Bird Color Space PC1 + Snake Color Space PC1 + Relative Maximum Bite Force	7	-269.07	73.72	0.00
Habitat + SVL + Predator + Normalized Peak Velocity + Bird Color Space PC1 + Snake Color Space PC1 + Snake Color Space PC2 + Relative Maximum Bite Force	8	-257.69	85.10	0.00
Habitat + SVL + Predator + Normalized Peak Velocity + Bird Color Space PC1 + Snake Color Space PC1 + Snake Color Space PC2 + Relative Maximum Bite Force + Habitat*SVL	9	-245.16	97.63	0.00
Habitat + SVL + Predator + Normalized Peak Velocity + Bird Color Space PC1 + Snake Color Space PC1 + Snake Color Space PC2 + Relative Maximum Bite Force + Habitat*SVL + Habitat*Normalized Peak Velocity	10	-236.24	106.54	0.00
Habitat + SVL + Predator + Normalized Peak Velocity + Bird Color Space PC1 + Snake Color Space PC1 + Snake Color Space PC2 + Relative Maximum Bite Force + Habitat*SVL + Habitat*Normalized Peak Velocity + Habitat*Bird Color Space PC1	11	-224.64	118.14	0.00
Habitat + SVL + Predator + Normalized Peak Velocity + Bird Color Space PC1 + Snake Color Space PC1 + Snake Color Space PC2 + Relative Maximum Bite Force + Habitat*SVL + Habitat*Normalized Peak Velocity + Habitat*Bird Color Space PC1 + Habitat*Snake Color Space PC1	12	214.77	128.02	0.00
Habitat + SVL + Predator + Normalized Peak Velocity + Bird Color Space PC1 + Snake Color Space PC1 + Snake Color Space PC2 + Relative Maximum Bite Force + Habitat*SVL + Habitat*Normalized Peak Velocity + Habitat*Bird Color Space PC1 + Habitat*Snake Color Space PC1 + Habitat*Snake Color Space PC2	13	-204.52	138.26	0.00
Habitat + SVL + Predator + Normalized Peak Velocity + Bird Color Space PC1 + Snake Color Space PC1 + Snake Color Space PC2 + Relative Maximum Bite Force + Habitat*SVL + Habitat*Normalized Peak Velocity + Habitat*Bird Color Space PC1 + Habitat*Snake Color Space PC1 + Habitat*Snake Color Space PC2 + Habitat*Relative Maximum Bite Force	14	-188.56	154.22	0.00
Habitat + SVL + Predator + Normalized Peak Velocity + Bird Color Space PC1 + Snake Color Space PC1 +	15	-176.16	166.62	0.00

Journal of Experimental Biology • Supplementary information

Snake Color Space PC2 + Relative Maximum Bite Force + Habitat*SVL + Habitat*Normalized Peak Velocity + Habitat*Bird Color Space PC1 + Habitat*Snake Color Space PC1 + Habitat*Snake Color Space PC2 + Habitat*Relative Maximum Bite Force + Predator*SVL				
Habitat + SVL + Predator + Normalized Peak Velocity + Bird Color Space PC1 + Snake Color Space PC1 + Snake Color Space PC2 + Relative Maximum Bite Force + Habitat*SVL + Habitat*Normalized Peak Velocity + Habitat*Bird Color Space PC1 + Habitat*Snake Color Space PC1 + Habitat*Snake Color Space PC2 + Habitat*Relative Maximum Bite Force + Predator*SVL + Predator*Normalized Peak Velocity	16	-167.06	175.72	0.00
Habitat + SVL + Predator + Normalized Peak Velocity + Bird Color Space PC1 + Snake Color Space PC1 + Snake Color Space PC2 + Relative Maximum Bite Force + Habitat*SVL + Habitat*Normalized Peak Velocity + Habitat*Bird Color Space PC1 + Habitat*Snake Color Space PC1 + Habitat*Snake Color Space PC2 + Habitat*Relative Maximum Bite Force + Predator*SVL + Predator*Normalized Peak Velocity + Predator*Bird Color Space PC1	17	-155.12	187.66	0.00
Habitat + SVL + Predator + Normalized Peak Velocity + Bird Color Space PC1 + Snake Color Space PC1 + Snake Color Space PC2 + Relative Maximum Bite Force + Habitat*SVL + Habitat*Normalized Peak Velocity + Habitat*Bird Color Space PC1 + Habitat*Snake Color Space PC1 + Habitat*Snake Color Space PC2 + Habitat*Relative Maximum Bite Force + Predator*SVL + Predator*Normalized Peak Velocity + Predator*Bird Color Space PC1 + Predator*Snake Color Space PC1	18	-144.89	197.90	0.00
Habitat + SVL + Predator + Normalized Peak Velocity + Bird Color Space PC1 + Snake Color Space PC1 + Snake Color Space PC2 + Relative Maximum Bite Force + Habitat*SVL + Habitat*Normalized Peak Velocity + Habitat*Bird Color Space PC1 + Habitat*Snake Color Space PC1 + Habitat*Snake Color Space PC2 + Habitat*Relative Maximum Bite Force + Predator*SVL + Predator*Normalized Peak Velocity + Predator*Bird Color Space PC1 + Predator*Snake Color Space PC1 + Predator*Snake Color Space PC2	19	-133.84	208.94	0.00
Habitat + SVL + Predator + Normalized Peak Velocity + Bird Color Space PC1 + Snake Color Space PC1 + Snake Color Space PC2 + Relative Maximum Bite Force + Habitat*SVL + Habitat*Normalized Peak Velocity + Habitat*Bird Color Space PC1 + Habitat*Snake Color Space PC1 + Habitat*Snake Color Space PC2 + Habitat*Relative Maximum Bite Force + Predator*SVL + Predator*Normalized Peak Velocity + Predator*Bird Color Space PC1 + Predator*Snake Color Space PC1 + Predator*Snake Color Space PC2 + Predator*Relative Maximum Bite Force	20	-117.33	225.45	0.00

Journal of Experimental Biology • Supplementary information

Table S6. AICc model selection for leaf-mimicking behaviors. Individual was treated as a random factor. Abbreviations as in Supplementary Table 3. Models with a ΔAICc <2 are denoted in bold.

Leaf-mimicking Models	K	AICc	ΔAICc	wi
**Habitat**	1	-288.21	**0.00**	1.00
Habitat + SVL	2	-275.50	12.71	0.00
Habitat + SVL + Normalized Peak Velocity	3	-266.81	21.40	0.00
Habitat + SVL + Normalized Peak Velocity + Relative Maximum Bite Force	4	-250.68	37.53	0.00
Habitat + SVL + Predator + Normalized Peak Velocity + Relative Maximum Bite Force	5	-242.92	45.29	0.00
Habitat + SVL + Predator + Normalized Peak Velocity + Bird Color Space PC1 + Relative Maximum Bite Force	6	-231.18	57.03	0.00
Habitat + SVL + Predator + Normalized Peak Velocity + Bird Color Space PC1 + Snake Color Space PC1 + Relative Maximum Bite Force	7	-221.27	66.94	0.00
Habitat + SVL + Predator + Normalized Peak Velocity + Bird Color Space PC1 + Snake Color Space PC1 + Snake Color Space PC2 + Relative Maximum Bite Force	8	-210.27	77.94	0.00
Habitat + SVL + Predator + Normalized Peak Velocity + Bird Color Space PC1 + Snake Color Space PC1 + Snake Color Space PC2 + Relative Maximum Bite Force + Habitat*SVL	9	-198.60	89.61	0.00
Habitat + SVL + Predator + Normalized Peak Velocity + Bird Color Space PC1 + Snake Color Space PC1 + Snake Color Space PC2 + Relative Maximum Bite Force + Habitat*SVL + Habitat*Normalized Peak Velocity	10	-190.94	97.27	0.00
Habitat + SVL + Predator + Normalized Peak Velocity + Bird Color Space PC1 + Snake Color Space PC1 + Snake Color Space PC2 + Relative Maximum Bite Force + Habitat*SVL + Habitat*Normalized Peak Velocity + Habitat*Bird Color Space PC1	11	-180.24	107.97	0.00
Habitat + SVL + Predator + Normalized Peak Velocity + Bird Color Space PC1 + Snake Color Space PC1 + Snake Color Space PC2 + Relative Maximum Bite Force + Habitat*SVL + Habitat*Normalized Peak Velocity + Habitat*Bird Color Space PC1 + Habitat*Snake Color Space PC1	12	-171.56	116.65	0.00
Habitat + SVL + Predator + Normalized Peak Velocity + Bird Color Space PC1 + Snake Color Space PC1 + Snake Color Space PC2 + Relative Maximum Bite Force + Habitat*SVL + Habitat*Normalized Peak Velocity + Habitat*Bird Color Space PC1 + Habitat*Snake Color Space PC1 + Habitat*Snake Color Space PC2	13	-161.70	126.51	0.00
Habitat + SVL + Predator + Normalized Peak Velocity + Bird Color Space PC1 + Snake Color Space PC1 + Snake Color Space PC2 + Relative Maximum Bite Force + Habitat*SVL + Habitat*Normalized Peak Velocity + Habitat*Bird Color Space PC1 + Habitat*Snake Color Space PC1 + Habitat*Snake Color Space PC2 + Habitat*Relative Maximum Bite Force	14	-146.26	141.95	0.00
Habitat + SVL + Predator + Normalized Peak Velocity + Bird Color Space PC1 + Snake Color Space PC1 +	15	-134.21	154.00	0.00

Snake Color Space PC2 + Relative Maximum Bite Force + Habitat*SVL + Habitat*Normalized Peak Velocity + Habitat*Bird Color Space PC1 + Habitat*Snake Color Space PC1 + Habitat*Snake Color Space PC2 + Habitat*Relative Maximum Bite Force + Predator*SVL				
Habitat + SVL + Predator + Normalized Peak Velocity + Bird Color Space PC1 + Snake Color Space PC1 + Snake Color Space PC2 + Relative Maximum Bite Force + Habitat*SVL + Habitat*Normalized Peak Velocity + Habitat*Bird Color Space PC1 + Habitat*Snake Color Space PC1 + Habitat*Snake Color Space PC2 + Habitat*Relative Maximum Bite Force + Predator*SVL + Predator*Normalized Peak Velocity	16	-126.16	162.05	0.00
Habitat + SVL + Predator + Normalized Peak Velocity + Bird Color Space PC1 + Snake Color Space PC1 + Snake Color Space PC2 + Relative Maximum Bite Force + Habitat*SVL + Habitat*Normalized Peak Velocity + Habitat*Bird Color Space PC1 + Habitat*Snake Color Space PC1 + Habitat*Snake Color Space PC2 + Habitat*Relative Maximum Bite Force + Predator*SVL + Predator*Normalized Peak Velocity + Predator*Bird Color Space PC1	17	-115.08	173.14	0.00
Habitat + SVL + Predator + Normalized Peak Velocity + Bird Color Space PC1 + Snake Color Space PC1 + Snake Color Space PC2 + Relative Maximum Bite Force + Habitat*SVL + Habitat*Normalized Peak Velocity + Habitat*Bird Color Space PC1 + Habitat*Snake Color Space PC1 + Habitat*Snake Color Space PC2 + Habitat*Relative Maximum Bite Force + Predator*SVL + Predator*Normalized Peak Velocity + Predator*Bird Color Space PC1 + Predator*Snake Color Space PC1	18	-105.98	182.23	0.00
Habitat + SVL + Predator + Normalized Peak Velocity + Bird Color Space PC1 + Snake Color Space PC1 + Snake Color Space PC2 + Relative Maximum Bite Force + Habitat*SVL + Habitat*Normalized Peak Velocity + Habitat*Bird Color Space PC1 + Habitat*Snake Color Space PC1 + Habitat*Snake Color Space PC2 + Habitat*Relative Maximum Bite Force + Predator*SVL + Predator*Normalized Peak Velocity + Predator*Bird Color Space PC1 + Predator*Snake Color Space PC1 + Predator*Snake Color Space PC2	19	-95.72	192.50	0.00
Habitat + SVL + Predator + Normalized Peak Velocity + Bird Color Space PC1 + Snake Color Space PC1 + Snake Color Space PC2 + Relative Maximum Bite Force + Habitat*SVL + Habitat*Normalized Peak Velocity + Habitat*Bird Color Space PC1 + Habitat*Snake Color Space PC1 + Habitat*Snake Color Space PC2 + Habitat*Relative Maximum Bite Force + Predator*SVL + Predator*Normalized Peak Velocity + Predator*Bird Color Space PC1 + Predator*Snake Color Space PC1 + Predator*Snake Color Space PC2 + Predator*Relative Maximum Bite Force	20	-79.85	208.36	0.00

Journal of Experimental Biology • Supplementary information
